# Seeking informal and formal help for mental health problems in the community: a secondary analysis from a psychiatric morbidity survey in South London

**DOI:** 10.1186/s12888-014-0275-y

**Published:** 2014-10-08

**Authors:** June SL Brown, Sara Evans-Lacko, Lisa Aschan, Max J Henderson, Stephani L Hatch, Matthew Hotopf

**Affiliations:** Psychology Department (PO77), Kings College London, Institute of Psychiatry, De Crespigny Park, London, SE5 8AF UK; Health Services and Population Research Department, (PO29), Institute of Psychiatry, De Crespigny Park, London, SE5 8AF UK; Department of Psychological Medicine, Weston Education Centre, Institute of Psychiatry, Cutcombe Road, London, SE5 9RJ UK

**Keywords:** Informal help, Formal help-seeking, Depression, Functioning, Friends, Family, Community psychiatric survey, Mental health

## Abstract

**Background:**

Only 30-35% of people with mental health problems seek help from professionals. Informal help, usually from friends, family and religious leaders, is often sought but is under-researched. This study aimed to contrast patterns of informal and formal help-seeking using data from a community psychiatric morbidity survey (n=1692) (South East London Community Health (SELCOH) Study).

**Methods:**

Patterns of help-seeking were analysed by clinical, sociodemographic and socioeconomic indicators. Factors associated with informal and formal help-seeking were investigated using logistic regression. Cross-tabulations examined informal help-seeking patterns from different sources.

**Results:**

‘Cases’ (n = 386) were participants who had scores of ≥ 12 on the Revised Clinical Interview Schedule (CIS-R), indicating a common mental disorder. Of these, 40.1% had sought formal help, (of whom three-quarters (29%) had also sought informal help), 33.6% had sought informal help only and only 26.3% had sought no help. When controlling for non-clinical variables, severity, depression, suicidal ideas, functioning and longstanding illnesses were associated with formal rather than informal help-seeking. Age and ethnic group influenced sources of informal help used. Younger people most frequently sought informal help only whereas older people tended to seek help from their family. There were ethnic group differences in whether help was sought from friends, family or religious leaders.

**Conclusions:**

This study has shown how frequently informal help is used, whether in conjunction with formal help or not. Among the ‘cases’, over 60% had sought informal help, whether on its own or together with formal help. Severity was associated with formal help-seeking. Patterns of informal help use have been found. The use and effectiveness of informal help merit urgent research.

## Background

It has been consistently found that only a third of individuals with diagnosable mental health problems seek formal help from health service providers [1-3] despite the availability of effective treatments [1]. The role of informal help from friends, families or other non-medical sources has been much less frequently researched.

Friends and family as well as religious leaders, or other non-health professionals usually offer informal help. It can also include self-help with other people with similar problems. Members of the public have been found to rate the helpfulness of informal help from friends and family more highly than that of professionals ([4,5]). Informal help is more difficult to evaluate because it happens more spontaneously and therefore studies are limited. Interestingly, the World Health Organisation (WHO) assert that primary care services should be supported by self-care and informal community care in their optimal mix of services [6].

Kleinman [7] argues that families, friends and other community leaders as well as ‘folk healers’ have historically played and still play an important role in how people perceive and deal with illness or disease. The social distance between the person with the problem and the informal helper is usually less great so that there is greater agreement about the perception of the problem and how it might be handled. Similarly, Kirmayer [8] argues that how mental health services are provided to diverse groups is becoming even more important, particularly with increasing globalization. In particular, the present model is very medicalised and based on western concepts of ‘illness’. Because of different uses of informal care by the different ethnic minority groups, it is argued that it will be important for services to be more ‘culturally competent’ [9].

There have been very few community surveys examining the use of formal and informal help, that is help from family, friends and spiritual or religious leaders. There have been a large number of studies on informal help but they have focused on specific disadvantaged groups, such as gay men with HIV [10] or partner abuse [11] or demographic groups such as young people [12], and ethnic minority groups [13,14]. Seeking help from multiple sources has also been found [13].

In the only community study in the UK to date investigating informal help among adults, Oliver et al. [15] found that 63.1% of 10302 participants preferred to seek help from friends and family when they were feeling ‘stress and strain’. Using the General Health Questionnaire (GHQ) [16] to measure severity, they found no differences in problem severity amongst those seeking informal help, but found differences with formal help, with 14% with less severe problems having sought formal help compared to 28% with more severe problems. In a smaller study using a psychiatric interview to assess severity, Rudell and colleagues [14] also found that informal help was commonly used, with talking to friends and family and keeping busy the most common strategies used.

Relatively little is known about its determinants and its effectiveness. Further, it is not clear where informal help fits into the current system of care. It may be that it is used as a precursor to formal help, or alternatively, it may be used alongside formal help. On the other hand, there is also some evidence that informal help prevents access to formal help, such that evidence-based treatments are not utilized. Lamb et al. [17] found that the low access to formal help of ‘hard to reach’ groups such as black and minority ethnic groups and depressed elderly people, was partly explained by these groups perceiving their problems as rooted in social problems and attempting to manage their problems themselves. They often sought help from close family and became isolated from other networks, rather than seeking formal help.

The characteristics of individuals who seek formal help are better understood. While severity of mental health problems is the most consistent predictor of formal help-seeking [18] other clinical variables have also been found to be relevant including functioning [18], perceived need [19] as well as co-morbidity [2]. Sociodemographic characteristics are also associated with help-seeking, with men more reluctant than women to seek formal help [18]. Ethnic differences have also been found with Asians tending to present less frequently in primary care settings even when controlling for severity [18] and GPs being less good at detecting the mental health problems of black Caribbean people [20]. People with diagnoses of depression have been found to be most likely to seek formal help compared with other mental disorders [18].

The aim of this study therefore is to investigate factors associated with informal help-seeking for mental health problems and contrast these to correlates of formal help-seeking using data from a community survey. The factors were socio-demographic, economic and clinical indicators. We also sought to explore the type of informal help people used.

## Method

### Design

We analysed data from a cross-sectional study of mental and physical health: the South East London Community Health (SELCoH) Study.

### Hypotheses

We set out to test the following hypotheses:

1. Compared to those who use formal help, exclusive use of informal help would be associated with less severe mental disorder.
2. Compared to those who use formal help, exclusive use of informal help would be associated with higher social support.
3. There would be sociodemographic factors (age, gender and ethnic group) with younger, female and black and ethnic minority groups being more likely to seek informal help.
4. There would be socioeconomic differences in help-seeking patterns with lower SES groups (characterised by low income and no qualifications) being more likely to seek formal help compared to informal help only.

### Setting and study participants

The South East London Community Health (SELCoH) study is a community survey of psychiatric and physical morbidity of 1698 adults, aged 16 years and over from 1075 randomly selected households in South London boroughs of Southwark and Lambeth. Data were collected between 2008–2010, applying similar methods to the British National Psychiatric Morbidity Surveys [21]; study methods are described in detail elsewhere [22,23].

In the two boroughs, there is higher deprivation than the England average, but similar proportions of economically active and inactive residents to greater London [24,25]. The boroughs are ethnically diverse, with a greater number of Black Caribbean and Black African residents but fewer South Asian residents than other areas of London [26,27]. The achieved SELCoH study sample was representative of the catchment area with regard to 2011 UK census demographic and socioeconomic indicators, with the exception of the study sample being slightly younger and including more students among the economically inactive (42.0% vs 33.3%).

Ethical approval was not sought for this study because we were performing a secondary analysis of data that had already been collected. The original study had received approval from the King’s College London research ethics committee, reference CREC/07/08-152.

### Measures

#### Dependent variables: use of formal and/or informal help in past year

Help-seeking within the past year was determined by self-report. ‘Formal help-seeking’ was tapped by a question: “In the past 12 months, have you spoken to a GP or family doctor, a psychological therapist/counsellor or other sources of help on your own behalf, either in person or by telephone about being anxious or depressed or a mental, nervous or emotional problem?”

Informal help-seeking was gauged by responses to: “In the past 12 months have you gone and seen any of the following for an emotional problem? Options included friends, family members, or spiritual/religious leaders”.

Because of the overlap of informal and formal help-seeking, we used four mutually exclusive help-seeking categories - no help, informal help only, both informal and formal help, or formal help only. In the regression analysis, the no help group was excluded and the three help-seeking groups were collapsed into informal help only and contrasted with formal help (with and without informal help).

#### Potential predictors of help-seeking

Clinical and non-clinical characteristics were investigated. Clinical variables examined included psychiatric severity, psychiatric diagnoses, suicidal indicators, longstanding illness and functioning indicators. ‘Non-clinical’ variables included sociodemographic characteristics, socioeconomic characteristics, and social support.

### Clinical variables

#### Psychiatric symptoms and diagnosis

##### Revised Clinical Interview Schedule (CIS-R)

The CIS-R [28] is a structured interview assessing psychiatric symptom status during the past month, and was used to assess severity of mental disorder. A total CIS-R score of 12 or above is conventionally used to indicate the presence of common mental disorder (CMD) (to be referred to as ‘cases’ for subsample analyses). We further categorized individuals scoring above the threshold into having severe CMD (18+), mild/moderate CMD (12–17) or being healthy (0–11) and used this measure as an independent variable.

The CIS-R provides ICD-10 diagnoses for ten psychiatric disorders through a standard algorithm. However, because of the very small numbers of people experiencing some disorders, only the four most common diagnoses were used for this study: depression (11.9%), non-specified neurotic disorder (6.63%), generalized anxiety disorder (3.51%) and phobia (1.73%).

#### Physical health and functioning

The global health item on the Short Form Health Survey SF-12 [29] was used to assess global health status. This item asked participants to rate their health on a five point scale from ‘poor’ to ‘excellent’. The variable was categorised as either Fair/poor vs Good/very good/excellent. We used two disability measures: functional limitations due to emotional health measured on the SF-12, and problems with activities in daily living (ADL) indicating limitations in five domains (personal help, transport, medical help, household activities and money). For these analyses, a cut-off of three ADL problems or more was used to indicate problems [18].

### Other clinical indicators

#### Suicidal ideation

Past-year suicidal ideation was measured through a single item question, replicating the measure from the Adult Psychiatric Morbidity Survey [21].

### Hazardous alcohol use

This was assessed through the Alcohol use Disorders Identification Test (AUDIT), a 10 item measure of alcohol consumption, dependence and misuse over the past year, with scores ranging from 0–40. Hazardous alcohol use was defined by scores of 8 or more [30].

### Past-year drug use

This was indicated through self-reported use of any of the following illicit drugs in the past year: cannabis, amphetamines, cocaine, ecstasy, LSD, tranquillizers, crack, and heroin.

### Long-standing illness

Participants were asked to report if they had any long-standing illness, disability or infirmity that had troubled or was likely to affect the participant over a period of time. The list included high blood pressure, bronchitis, heart trouble, cancer etc.

### Non-clinical variables

**Sociodemographic variables** included age, gender, ethnicity, relationship status, and migrant status.

Age was measured continuously and also categorized into 5 groups. Ethnicity was categorised into 5 groups. Current relationship status was categorized as married/cohabiting vs. not. Migrant status indicated whether or not the person was born in the UK.

**Socioeconomic indicators** included education, employment status and household income. There were 4 employment categories and 4 educational categories. Income was measured as the gross annual household income from all sources before any deductions, and categorized into 5 categories.

**Social support** Presence of emotional social support was indicated by 2 items: having someone to talk to about something that was bothering you or when you felt lonely and wanted some company; and having someone who makes you feel good, loved or cared for.

### Analysis

All analyses were carried out in Stata 11 and accounted for household clustering and non-response using survey weights and applying ‘svy’ commands in order to generate robust standard errors [31]. The prevalence of informal and formal and sources of informal help were estimated within the full sample and within ‘cases’. The prevalence of informal sources of help was also estimated within the subsample of informal help users.

In generating the four mutually exclusive utilisation categories (no help, informal help only, both informal and formal, only formal), the sample size was reduced from 1698 to 1610 due to missing observations (86 true missing from the informal help item; two refusals to answer the formal help question). Within this sample, percentage prevalence estimates of informal and formal help use were described by sociodemographic, socio-economic and clinical variables using Pearson’s χ^2^ test with Rao & Scott corrections to test for differences.

Unadjusted and fully adjusted logistic regression analyses were carried out in order to estimate factors associated with using informal help only versus any formal help (including combined informal and formal help). Adjusted estimations of socio-demographic and SES associations controlled for each other without adjusting for health related indicators or social support. Where clinical/health related and social support indicators were the independent variables of interest, they were entered separately from each other, only controlling for sociodemographics and SES to avoid collinearity. These models adjusted for age using the continuous measure, rather the categorical measure used to adjust for sociodemographic and socio-economic associations. All of the adjusted models were tested for goodness-of-fit using the post-hoc ‘svylogitgof’ command, which is appropriate for survey data [32].

Finally, we described use of specific sources of informal help (i.e., family, friend, religious leader, other) by gender, age, ethnicity and migrant status by calculating percentage prevalence estimates.

## Results

### Informal and formal help-seeking patterns

#### Participant characteristics

Table 1 shows the prevalence of the different types of help seeking.

**Table 1:** **Prevalence of help seeking**

	**Full sample (N = 1698)**			**CIS-R ≥12 (n = 396)**			**Informal help users (n = 577)**		
	**n**	**%**	**(95% CI)**	**n**	**%**	**(95% CI)**	**n**	**%**	**(95% CI)**
Type of help*									
Overall formal help	290	17.5	(15.7-19.5)	156	39.4	(34.4-44.5)		-	
Overall informal help	579	36.1	(33.5-38.7)	239	62.5	(57.2-67.5)		-	
**Help composite variable**									
Formal help only	88	5.6	(4.5-7.0)	42	11.1	(8.2-15.0)		-	
Informal help and formal help	201	12.8	(11.2-14.6)	113	29.0	(24.6-33.7)		-	
Informal help only	377	23.3	(21.1-25.6)	126	33.6	(28.8-38.8)		-	
No help	944	58.3	(55.7-61.0)	105	26.3	(21.9-31.4)		-	
**Informal help use**									
Friend	397	24.1	(21.9-26.5)	160	40.1	(35.1-45.4)	397	67.0	(62.7-71.1)
Family	370	23.4	(21.2-25.7)	158	42.1	(36.8-47.5)	370	64.9	(60.7-68.9)
Religious leaders	27	1.9	(1.2-2.8)	13	3.3	(1.8-5.8)	27	5.2	(3.5-7.8)
Other	42	2.4	(1.8-3.3)	14	3.1	(1.8-5.3)	42	6.6	(4.9-9.0)

Frequencies show actual counts; percentages have been weighted.

p-values show significance level of Pearson’s Chi square test with Rao & Scott corrections.

*with overlaps of formal and informal help.

For the sample as a whole, informal help was sought twice as frequently (36.1%) as formal help (17.5%). Of those who sought formal help, the majority used informal help as well (69.3%), whereas most people who sought informal help did not use formal help (65.1%).

Among ‘cases’ (n = 386), 33.6% had sought informal help only. Of the 40.1% of ‘cases’ who had sought formal help, three-quarters (29%) had also sought informal help, meaning only 11.1% sought formal help alone. Only 26.3% had sought no help. The most frequent form of informal help used was from friends or family with a small minority consulting religious leaders.

Sociodemographic and socio-economic patterns for the 4 help-seeking groups are described in Table 2. Men were less likely to seek help than women, the differences being particularly pronounced with informal help. Younger people more frequently sought informal help only. The 56 and older group sought no help most frequently, and they used a slightly higher proportion of formal help to informal help. The 26–40 (19.7%) and 41–55 (22.2%) year old age groups sought formal help most frequently, whether in combination with informal help or not.

**Table 2:** **The socio-demographic and socio-economic distribution of formal and informal help seeking (N = 1,610)**

		**Formal only (n = 88)**			**Formal and informal (n = 377)**			**Informal only (n = 201)**			**No help (n = 944)**				
	**N**	**n**	**%**	**(95% CI)**	**n**	**%**	**(95% CI)**	**n**	**%**	**(95% CI)**	**n**	**%**	**(95% CI)**	**Χ** ^**2**^	***p***
***Socio-demographic***															
*Gender*														11.63	<0.001
Male	702	36	5.2	(3.7-7.2)	65	8.9	(7.0-11.3)	130	17.9	(15.3-20.9	471	67.9	(64.3-71.3)		
Female	908	52	5.8	(4.4-7.6)	136	14.7	(12.5-17.2)	247	25.9	(23.0-29.0)	473	53.6	(50.1-57.0)		
*Age*														4.09	<0.001
16-25	387	16	4.2	(2.6-6.7)	43	11.8	(8.7-15.8)	108	28.8	(24.3-33.6)	220	55.2	(50.1-60.2)		
26-40	521	24	4.6	(3.1-6.9)	76	15.1	(12.2-18.7)	141	28.1	(24.2-32.4)	280	52.1	(47.5-56.6)		
41-55	398	29	7.4	(5.1-10.4)	55	14.8	(11.5-18.9)	80	20.7	(16.9-25.1)	234	57.1	(52.0-62.1)		
56 or older	304	19	6.2	(3.9-9.6)	27	9.4	(6.5-13.5)	48	16.5	(12.5-21.3)	210	68.0	(62.1-73.3)		
*Ethnic group*														1.10	0.356
White	987	58	6.1	(4.7-7.9)	133	13.5	(11.3-15.9)	231	23.2	(20.5-26.2)	565	57.2	(53.8-60.6)		
Black Caribbean	137	11	8.0	(4.4-14.2)	14	11.1	(6.7-17.8)	34	24.1	(17.0-32.9)	78	56.8	(47.8-65.5)		
Black African	225	11	4.7	(2.6-8.4)	21	10.3	(6.7-15.6)	46	19.2	(14.5-25.0)	147	65.7	(58.6-72.2)		
Asian	60	0	-		7	12.9	(6.3-24.5)	20	33.7	(21.9-48.0)	33	53.4	(40.1-66.3)		
Other	199	8	4.0	(2.0-7.9)	25	12.8	(8.8-18.3)	45	23.8	(17.9-30.9)	121	59.4	(52.1-66.4)		
*Relationship status*														4.48	0.004
Married/cohabitating	739	32	4.7	(3.3-6.7)	79	11.0	(8.8-13.6)	154	20.8	(17.9-24.0)	474	63.5	(59.6-67.2)		
Non-married/non-cohabitating*	871	56	6.4	(4.9-8.3)	122	14.3	(12.0-17.0)	233	25.3	(22.3-28.6)	470	54.0	(50.3-57.6)		
*Migration status*														2.07	0.102
Non-migrant	970	56	6.0	(4.6-7.8)	120	12.4	(10.4-14.8)	206	21.4	(18.8-24.3)	588	60.2	(56.8-63.5)		
Migrant	633	29	4.6	(3.2-6.6)	81	13.5	(10.9-16.6)	170	26.2	(22.8-30.0)	353	55.7	(51.4-59.9)		
***Socio-economic***															
*Employment status*														3.03	0.001
Employed	863	38	4.6	(3.3-6.2)	87	10.5	(8.5-12.8)	210	24.5	(21.6-27.7)	528	60.4	(56.9-63.9)		
Unemployed	164	16	10.2	(6.2-16.2)	31	18.9	(13.5-25.7)	41	25.4	(19.0-33.1)	76	45.6	(37.8-53.5)		
Student	239	12	4.9	(2.7-8.7)	28	12.7	(8.8-18.0)	63	26.9	(21.5-33.1)	136	55.5	(48.9-61.9)		
Other	336	22	6.4	(4.2-9.8)	53	14.8	(11.3-19.1)	61	18.2	(14.2-23.0)	200	60.6	(54.9-65.9)		
*Education*														1.90	0.049
No qualifications	230	16	6.5	(3.9-10.7)	28	12.0	(8.2-17.2)	37	15.9	(11.5-21.6)	149	65.6	(58.8-71.8)		
GCSE	318	24	7.9	(5.2-11.7)	43	13.7	(10.2-18.2)	78	24.8	(20.2-30.0)	173	53.6	(47.9-59.3)		
A-level	407	23	5.7	(3.8-8.6)	53	13.5	(10.2-17.6)	100	25.1	(20.9-29.8)	231	55.7	(50.6-60.7)		
Degree or above	655	25	4.0	(2.7-5.9)	77	12.2	(9.8-15.1)	162	24.7	(21.5-28.3)	391	59.1	(55.1-63.0)		
*Annual household income*														2.38	0.005
£0-5,475	133	11	7.1	(3.9-12.6)	36	27.3	(19.8-36.3)	20	16.1	(10.3-24.4)	66	49.5	(40.4-58.6)		
£5,476-12,097	201	14	6.6	(3.8-11.2)	31	15.1	(10.7-21.1)	48	23.2	(17.7-29.9)	108	55.0	(47.7-62.1)		
£12,098-20,753	196	7	4.0	(1.9-8.4)	23	12.2	(8.1-17.9)	49	24.5	(18.8-31.4)	117	59.3	(51.8-66.3)		
£20,754-31,494	167	11	6.4	(3.5-11.3)	16	9.9	(6.1-15.7)	43	25.2	(18.9-32.8)	97	58.5	(50.4-66.2)		
£31,495 or more	662	30	4.7	(3.3-6.7)	75	11.6	(9.3-14.4)	156	23.6	(20.4-27.1)	401	60.1	(56.2-63.9)		

Frequencies show actual counts; percentages have been weighted.

Counts may not add up due to missing values.

χ^2^ statistics and *p*-values are weighted outcomes from Pearson’s Chi square tests with Rao & Scott corrections.

*Non-married/cohabitating category include single, divorced/separated, and widowed relationship status.

There were no significant differences by migrant status or ethnicity. Relationship status differentiated the groups. Non-married or non-cohabiting participants were more likely to seek formal and/or informal help (20.7%) or informal help only (25.3%).

In terms of socio-economic differences, the unemployed group was much more likely to seek formal – as well as informal – help (total 54.5%) than the other employment groups whereas employed participants tended to seek informal help only (24.5%) or no help (60.4%). Marginally significant differences across education qualification levels were found. Those with no qualifications tended to be less likely to seek any form of help, particularly informal help on its own. Income differences were significant, with the lowest income group being most likely to seek formal help (with or without informal help) but made less use of informal help on its own than other groups.

Clinical differences across the 4 help-seeking groups are shown in Table 3. Participants scoring above the threshold on the CIS-R (those categorized as 12–17 or 18+) were more likely than those below the threshold to seek formal help, whether on its own or with informal help. Compared to those with scores below 12, they were more likely to seek informal help only and less likely to seek no help. While those with CIS-R scores below 12 were proportionately least likely to seek any help, 45 (3.8%) had sought formal help. Looking at it another way, of the 88 individuals who had sought formal help only, 45 (51.1%) scored below the threshold. Similarly, of the 201 who had sought both informal and formal help, 88 (43.8%) were below the threshold.

**Table 3:** **The distribution of formal and informal help seeking by health and social support indicators (N = 1,610)**

		**Formal only (n = 88)**			**Formal and informal (n = 201)**			**Informal only (n = 377)**			**No help (n = 944)**				
	**N**	**n**	**%**	**(95% CI)**	**n**	**%**	**(95% CI)**	**n**	**%**	**(95% CI)**	**n**	**%**	**(95% CI)**	**Χ** ^**2**^	**p**
***Clinical indicators***															
**CMD (CIS-R score)**														40.20	<0.001
No CMD (<12)	1,219	45	3.8	(2.8-5.1)	88	7.5	(6.0-9.2)	250	19.9	(17.6-22.3)	836	68.9	(66.0-71.6)		
CMD (12–17)	188	17	9.5	(5.8-15.0)	39	21.8	(16.3-28.5)	66	35.9	(29.0-43.5)	66	32.9	(26.1-40.5)		
Symptoms likely to require treatment (≥18)	198	25	12.6	(8.5-18.2)	74	35.5	(29.1-42.6)	60	31.4	(25.1-38.5)	39	20.4	(15.1-27.0)		
**Any CIS-R primary diagnosis**														74.81	<0.001
No	1,164	44	4.0	2.9 5.4	75	6.8	(5.4-8.6)	231	19.2	(16.9-21.7)	814	70.0	(67.1-72.7)		
Yes	442	43	9.6	7.1 13.0	126	28.0	(24.0-32.4)	145	33.5	(29.1-38.3)	128	28.8	(24.5-33.6)		
**Non-specified neurotic disorder**														5.11	0.002
No	1,500	81	5.6	4.5 6.9	185	12.6	(10.9-14.5)	336	22.2	(20.1-24.6)	898	59.6	(56.8-62.3)		
Yes	106	6	6.0	2.7 13.0	16	15.4	(9.6-23.7)	40	37.8	(28.5-48.0)	44	40.9	(31.4-51.1)		
**Generalised anxiety disorder**														8.56	<0.001
No	1,533	78	5.2	4.2 6.6	183	12.2	(10.6-14.1)	352	22.8	(20.6-25.2)	920	59.7	(57.0-62.4)		
Yes	73	9	12.8	6.5 23.9	18	24.7	(15.7-36.5)	24	32.3	(22.4-44.0)	22	30.2	(20.5-42.1)		
**Phobia (any)**														2.83	0.037
No	1,559	86	5.7	4.6 7.1	190	12.5	(10.9-14.4)	359	23.0	(20.8-25.3)	924	58.8	(56.1-61.5)		
Yes	47	1	2.3	0.3 14.6	11	22.5	(12.7-36.7)	17	33.7	(21.5-48.4)	18	41.6	(27.7-56.9)		
**Depression**														52.28	<0.001
No	1,417	61	4.6	3.5 5.9	131	9.5	(8.0-11.3)	325	22.5	(20.2-24.9)	900	63.4	(60.7-66.1)		
Yes	189	26	12.9	8.8 18.5	70	35.9	(29.4-43.0)	51	28.9	(22.6-36.1)	42	22.2	(16.7-28.9)		
**Other** ^**†**^														12.40	<0.001
No	1,579	86	5.6	4.5 7.0	190	12.4	(10.7-14.2)	363	22.7	(20.6-25.0)	940	59.3	(56.6-61.9)		
Yes	27	1	3.8	0.5 22.6	11	37.2	(21.2-56.6)	13	53.0	(34.1-71.1)	2	6.0	(1.4-21.7)		
**Suicidal ideation**														21.53	<0.001
No	1,518	77	5.2	(4.2-6.6)	164	11.2	(9.6-13.1)	363	23.8	(21.5-26.2)	914	59.7	(57.0-62.5)		
Yes	84	11	13.0	(7.2-22.3)	36	39.6	(29.5-50.7)	12	14.7	(8.3-24.8)	25	32.6	(23.0-44.0)		
**Hazardous alcohol use**														4.50	0.002
No	1,272	66	5.4	(4.3-6.9)	146	11.9	(10.1-14.0)	290	22.1	(19.8-24.7)	770	60.5	(57.5-63.4)		
Yes	330	22	6.6	(4.2-10.2)	54	16.6	(13.0-21.0)	85	28.6	(23.7-34.1)	169	48.2	(42.4-53.9)		
**Drug use (past year)**														8.96	<0.001
No	1,261	66	5.5	(4.3-7.0)	142	11.7	(9.9-13.7)	273	21.5	(19.2-24.1)	780	61.3	(58.4-64.2)		
Yes	345	22	6.5	(4.1-9.9)	58	17.5	(13.7-22.1)	103	31.0	(26.1-36.4)	162	45.0	(39.5-50.6)		
**Long-standing illness**														10.12	<0.001
No	959	42	4.8	(3.5-6.5)	82	8.6	(7.0-10.6)	238	24.8	(22.0-27.8)	597	61.7	(58.4-64.9)		
Yes	643	46	6.7	(4.9-8.9)	117	17.6	(14.7-20.9)	137	21.4	(18.2-24.9)	343	54.3	(50.1-58.5)		
**Self-rated health**														20.22	<0.001
Good or better	1,314	59	4.7	(3.6-6.0)	131	10.0	(8.4-11.8)	303	22.7	(20.4-25.2)	821	62.7	(59.8-65.5)		
Fair or poor	288	28	9.4	(6.5-13.4)	68	23.7	(19.0-29.1)	73	25.8	(20.9-31.5)	119	41.1	(35.2-47.3)		
**ADL problems with:**															
*Personal help*														3.69	0.012
No	1,531	84	5.7	(4.6-7.1)	183	12.1	(10.4-13.9)	359	23.3	(21.1-25.7)	905	58.9	(56.2-61.6)		
Yes	61	3	4.2	(1.3-12.3)	16	26.4	(16.5-39.5)	14	24.0	(14.5-37.1)	28	45.4	(32.7-58.7)		
*Using transport*														6.60	<0.001
No	1,508	76	5.1	(4.0-6.4)	179	12.1	(10.4-13.9)	353	23.2	(21.0-25.6)	900	59.6	(56.8-62.3)		
Yes	84	11	13.0	(7.1-22.4)	20	22.8	(14.8-33.6)	20	24.8	(16.4-35.6)	33	39.4	(29.2-50.7)		
*Medical help*														1.86	0.134
No	1,567	87	5.7	(4.6-7.1)	193	12.5	(10.9-14.4)	365	23.1	(21.0-25.5)	922	58.6	(55.9-61.3)		
Yes	25	0	-		6	24.7	(11.0-46.5)	8	31.9	(16.1-53.3)	11	43.4	(24.9-63.9)		
*Household activities*														9.62	<0.001
No	1,480	75	5.2	(4.1-6.6)	171	11.7	(10.0-13.5)	341	22.7	(20.5-25.1)	893	60.4	(57.7-63.1)		
Yes	112	12	10.0	(5.6-17.1)	28	24.5	(17.2-33.7)	32	29.8	(21.6-39.4)	40	35.8	(27.1-45.5)		
*Money*														5.20	0.001
No	1,496	76	5.3	(4.2-6.7)	183	12.4	(10.7-14.3)	342	22.7	(20.5-25.1)	895	59.6	(56.8-62.2)		
Yes	96	11	10.8	(5.9-19.2)	16	18.4	(11.2-28.7)	31	32.4	(23.4-43.1)	38	38.3	(28.4-49.3)		
**No. ADL problems**														4.63	0.003
<3	1,548	83	5.5	(4.4-6.9)	187	12.2	(10.6-14.0)	360	23.1	(20.9-25.4)	918	59.2	(56.5-61.9)		
≥3	44	4	7.9	(2.8-20.2)	12	28.5	(16.5-44.4)	13	30.3	(18.0-46.2)	15	33.4	(20.4-49.5)		
**Functional limits due**															
**to emotional health**														63.39	<0.001
No	1,303	56	4.4	(3.4-5.7)	105	8.4	(6.9-10.2)	272	20.5	(18.3-22.9)	870	66.7	(63.9-69.4)		
Yes	293	30	10.1	(7.1-14.3)	93	30.7	(25.6-36.4)	102	35.2	(29.6-41.3)	68	23.9	(19.1-29.4)		
***Social support***															
**Someone to talk to**														5.99	<0.001
No	113	13	13.0	(7.5-21.6)	8	6.3	(3.1-12.4)	18	15.5	(9.7-23.7)	74	65.2	(55.4-73.9)		
Yes	1,479	75	5.1	(4.1-6.4)	192	13.3	(11.6-15.3)	353	23.7	(21.5-26.2)	859	57.8	(55.0-60.5)		
**Someone to make you feel cared for**														1.30	0.273
No	94	10	10.0	(5.2-18.6)	14	14.3	(8.4-23.3)	17	19.6	(12.1-30.1)	53	56.1	(45.2-66.4)		
Yes	1,502	77	5.3	(4.2-6.6)	186	12.7	(11.0-14.6)	356	23.5	(21.3-25.9)	883	58.5	(55.8-61.3)		

Frequencies show actual counts; percentages have been weighted.

Counts may not add up due to missing values.

χ^2^ statistics and *p*-values are weighted outcomes from Pearson’s Chi square tests with Rao & Scott corrections.

^†^Other category includes obsessive compulsive disorder (n = 2), panic disorder (n = 8), and mixed anxiety and depressive disorder (n = 17).

CMD, Common Mental Disorder; CIS-R Clinical Interview Schedule revised; ADL, activities in daily life.

Individuals with diagnoses of depression and with suicidal ideation tended to be more likely to use formal help, with about half seeking formal help. Those with suicidal ideation were also significantly less likely to seek informal help only. Participants with long-standing illnesses tended to seek formal help, whether with or without informal help more often, and informal help alone less often, compared to those without these problems. Those reporting functional limitations due to emotional health and activities in daily living also indicated increased use of all types of help (informal help only, both informal and formal help, and formal help only). We found that those with someone to talk to tended to seek informal help rather than formal help. Conversely, those who did not have someone to talk to, tended to seek formal help. However, no differences were found between those who did and did not endorse the item about whether they had someone to make them feel cared for.

Table 4 describes results of the logistic regression and factors associated with exclusive informal help seeking versus those who sought formal help and gives the unadjusted and adjusted results. All adjusted models had acceptable goodness-of-fit (p > 0.05). The degrees of freedom for these tests were 9 and within the range of 465–8. The adjusted results show that those with CIS-R scores above the threshold, any primary diagnosis, a depression diagnosis, suicidal ideation, longstanding illnesses, functional limitations and poor perceived health were less likely to seek informal help, but seek formal help. Contrary to prediction, social support was not associated with exclusive informal help seeking.

**Table 4:** **Logistic regression analyses comparing informal (only) help users with formal help users (N = 666)**

		**Informal only**		**Unadjusted**			**Adjusted** ^**‡**^		
	**N**	**n**	**%**	**OR**	**(95% CI)**	***p***	**OR**	**(95% CI)**	***p***
***Socio-demographic and SES***									
**Gender**						0.991			0.958
Male	231	130	55.9	1.0			1.0		
Female	435	247	55.8	1.0	(0.7-1.4)		1.0	(0.7-1.5)	
**Age**						0.013			0.555
16-25	167	108	64.2	1.0			1.0		
26-40	241	141	58.7	0.8	(0.5-1.2)		0.7	(0.4-1.2)	
41-55	164	80	48.3	0.5	(0.3-0.8)		0.5	(0.3-0.9)	
56 or older	94	48	51.4	0.6	(0.3-1.0)		0.9	(0.5-1.9)	
**Ethnic group**						0.528			0.323
White	422	231	54.3	1.0			1.0		
Black Caribbean	59	34	55.7	1.1	(0.6-1.9)		0.8	(0.4-1.6)	
Black African	78	46	56.1	1.1	(0.6-1.8)		0.6	(0.3-1.2)	
Asian	27	20	72.4	2.2	(0.9-5.5)		1.3	(0.4-3.9)	
Other	78	45	58.7	1.2	(0.7-2.0)		1.4	(0.8-2.5)	
**Relationship status**						0.653			0.597
Married/cohabitating	265	154	56.9	1.0			1.0		
Non-married/cohabitating*	401	223	55.1	0.9	(0.7-1.3)		0.9	(0.6-1.3)	
**Migration status**						0.189			0.415
Non-migrant	382	206	53.8	1.0			1.0		
Migrant	280	170	59.2	1.2	(0.9-1.7)		1.2	(0.8-1.8)	
**Employment**						0.006			0.145
Employed	335	210	62.0	1.0			1.0		
Unemployed	88	41	46.6	0.5	(0.3-0.9)		0.8	(0.4-1.4)	
Student	103	63	60.4	0.9	(0.6-1.5)		0.7	(0.4-1.3)	
Other	136	61	46.2	0.5	(0.3-0.8)		0.5	(0.3-0.9)	
**Education**						0.022			0.910
No qualifications	81	37	46.1	0.6	(0.3-1.0)		0.9	(0.5-1.9)	
GCSE	145	78	53.5	0.8	(0.5-1.1)		1.0	(0.6-1.8)	
A-level	176	100	56.6	0.9	(0.6-1.3)		1.1	(0.7-1.8)	
Degree or above	264	162	60.4	1.0			1.0		
**Annual household income**						<0.001			0.176
£0-5,475	67	20	32.0	0.3	(0.2-0.6)		0.5	(0.2-1.0)	
£5,476-12,097	93	48	51.6	0.7	(0.4-1.2)		1.0	(0.5-1.9)	
£12,098-20,753	79	49	60.2	1.0	(0.6-1.8)		1.3	(0.7-2.4)	
£20,754-31,494	70	43	60.7	1.1	(0.6-1.9)		1.2	(0.6-2.1)	
£31,495 or more	261	156	59.2	1.0			1.0		
***Clinical indicators***									
**CMD (CIS-R score)**						<0.001			<0.001
No CMD (<12)	383	250	63.8	1.0			1.0		
CMD (12–18)	122	66	53.5	0.7	(0.4-1.0)		0.6	(0.4-1.0)	
Symptoms likely to require treatment (≥18)	159	60	39.5	0.4	(0.2-0.6)		0.4	(0.3-0.7)	
**Any CIS-R primary diagnosis**						<0.001			0.002
No	350	231	64.0	1.0			1.0		
Yes	314	145	47.1	0.5	(0.4-0.7)		0.6	(0.4-0.8)	
**Non-specified neurotic disorder**						0.223			0.252
No	602	336	55.0	1.0			1.0		
Yes	62	40	63.8	1.4	(0.8-2.6)		1.5	(0.7-3.0)	
**Generalised anxiety disorder**						0.175			0.344
No	613	352	56.6	1.0			1.0		
Yes	51	24	46.3	0.7	(0.4-1.2)		0.7	(0.4-1.4)	
**Phobia (any)**						0.847			0.855
No	635	359	55.7	1.0			1.0		
Yes	29	17	57.6	1.1	(0.5-2.3)		1.1	(0.4-2.8)	
**Depression**						<0.001			<0.001
No	517	325	61.4	1.0			1.0		
Yes	147	51	37.1	0.4	(0.3-0.5)		0.4	(0.2-0.6)	
**Other primary CIS-R diagnosis** ^**†**^						0.952			0.895
No	639	363	55.8	1.0			1.0		
Yes	25	13	56.4	1.0	(0.4-2.3)		0.9	(0.4-2.3)	
**Suicidal ideation**						<0.001			<0.001
No	604	363	59.0	1.0			1.0		
Yes	59	12	21.9	0.2	(0.1-0.4)		0.2	(0.1-0.4)	
**Hazardous alcohol use**						0.856			0.484
No	502	290	56.0	1.0			1.0		
Yes	161	85	55.2	1.0	(0.7-1.4)		1.2	(0.8-1.8)	
**Drug use (past year)**						0.882			0.879
No	481	273	55.7	1.0			1.0		
Yes	183	103	56.4	1.0	(0.7-1.5)		1.0	(0.7-1.6)	
**Long-standing illness**						<0.001			0.003
No	362	238	64.9	1.0			1.0		
Yes	300	137	46.8	0.5	(0.3-0.7)		0.5	(0.4-0.8)	
**No. ADL problems**						0.269			0.305
<3	630	360	56.5	1.0			1.0		
≥3	29	13	45.5	0.6	(0.3-1.4)		0.6	(0.2-1.6)	
**Self-rated health**						<0.001			0.024
Good or better	493	303	60.8	1.0			1.0		
Fair or poor	169	73	43.9	0.5	(0.4-0.7)		0.6	(0.4-0.9)	
**Functional limits due to emotional health**						<0.001			<0.001
No	433	272	61.5	1.0			1.0		
Yes	169	102	46.3	0.5	(0.4-0.8)		0.5	(0.3-0.8)	
***Social support***									
**Someone to talk to**						0.174			0.456
No	433	18	44.5	1.0			1.0		
Yes	225	353	56.3	1.6	(0.8-3.2)		1.4	(0.6-3.5)	
**Someone to make you feel cared for**						0.172			0.521
No	39	17	44.6	1.0			1.0		
Yes	620	356	56.6	1.6	(0.8-3.2)		1.4	(0.5-3.6)	

Frequencies show actual counts, percentages and regression analyses have been weighted.

Counts may not add up due to missing values.

p-values for age, education, income and CMD test for trends.

*Non-married/cohabitating category include single, divorced/separated, and widowed relationship status.

^†^Other category includes obsessive compulsive disorder (n = 2), panic disorder (n = 7), and mixed anxiety and depressive disorder (n = 16).

^‡^Socio-demographic and socio-economic indicators adjustfor each other, without including clinical indicators or social support variables in the model. Clinical indicators and social support variables are tested in separate models adjusting for all socio-demographic and SES indicators. These models use adjust for age using the continuous measure.

SES, socio-economic status; CMD, Common Mental Disorder; CIS-R Clinical Interview Schedule revised; ADL, activities in daily life.

Table 5 explores socio-demographic variables by source of informal help. When the pattern of informal help was examined, regardless of caseness, significant age differences were found. The 16–25 year olds were much more likely to seek help from friends (81.9%) whereas family members were used mainly by older people (70.5%). Religious leaders were most often used by those aged 41 and above (16.6%). Different patterns of informal and formal help-seeking were also shown according to ethnic groups. Black Caribbeans tended to seek help from friends (77.9%) and were less likely to use family members. In contrast, Asians tended to use family members (88.4%) but not friends. Religious leaders were most likely to be used by black Africans (17.8%), Asians (14.9%) and by migrants (8.7%).

**Table 5:** **The socio-demographic distribution of sources of help among informal help users (n = 577)**

		**Friend**				**Family**				**Religious leader**				**Other**			
	**N**	**n**	**%**	**(95% CI)**	***p***	**n**	**%**	**(95% CI)**	***p***	**n**	**%**	**(95% CI)**	***p***	**n**	**%**	**(95% CI)**	***p***
**Gender**					0.595				0.562				0.584				0.001
Male	194	130	65.3	(57.8-72.1)		121	63.0	(55.8-69.7)		9	4.4	(2.3-8.4)		24	12.6	(8.5-18.3)	
Female	383	267	67.6	(62.4-72.4)		249	65.5	(60.5-70.2)		18	5.5	(3.4-8.6)		18	4.7	(2.9-7.4)	
**Age**					<0.001				0.526				0.047				0.736
16-25	148		120	81.9	(74.7-87.4)		95	63.1	(54.0-71.2)		3	2.1	(0.7-6.4)		7	4.8	(2.3-9.8)
26-40	220	153	70.2	(63.8-75.9)		136	62.0	(55.7-67.9)		8	3.6	(1.7-7.7)		17	6.6	(4.1-10.4)	
41-55	134	86	62.0	(53.1-70.1)		86	66.2	(57.5-73.8)		9	6.6	(3.3-12.5)		12	8.2	(4.6-14.1)	
56 or older	75	38	50.6	(38.9-62.3)		53	70.5	(58.8-80.0)		7	10.0	(4.8-19.6)		6	7.0	(3.1-15.1)	
**Ethnic group**					0.004				0.040				<0.001				0.238
White	365	254	67.4	(61.7-72.6)		237	65.8	(60.4-70.8)		10	2.7	(1.4-5.2)		32	8.0	(5.6-11.2)	
Black Caribbean	48	37	77.9	(63.5-87.7)		26	53.6	(40.3-66.4)		2	3.5	(0.5-20.9)		3	4.3	(1.3-12.7)	
Black African	66	44	66.4	(53.5-77.2)		43	66.3	(52.8-77.6)		10	17.8	(9.4-31.3)		2	2.8	(0.7-10.7)	
Asian	26	9	32.6	(17.4-52.7)		22	88.4	(68.5-96.4)		3	14.9	(4.9-37.4)		3	11.1	(3.4-30.4)	
Other	70	52	72.0	(58.9-82.1)		41	58.3	(46.2-69.5)		2	5.1	(1.3-18.1)		2	3.1	(0.8-11.6)	
**Migration status**					0.662				0.882				0.004				0.203
Non-migrant	326	227	67.9	(62.0-73.3)		211	65.2	(59.6-70.5)		9	2.6	(1.2-5.4)		27	7.7	(5.2-11.1)	
Migrant	250	170	66.0	(59.5-72.0)		159	64.6	(58.3-70.5)		18	8.7	(5.3-13.9)		14	5.0	(2.9-8.5)	

Frequencies show actual counts; percentages have been weighted.

Counts may not add up due to missing values.

p-values show significance level of Pearson’s Chi square test with Rao & Scott corrections.

## Discussion

While determinants of formal help have been frequently researched, those of informal help have been under-researched. To our knowledge, this is the first time that predictors of informal help have been systematically investigated in a community survey of adults in the UK. We found that informal help is extremely commonly used among the whole sample as well as among ‘cases’. Among the ‘cases’, 62.6% had sought informal help, whether on its own (33.6%) or together with formal help (29%) with 26.3% not having sought any help at all. Only 11.1% had sought formal help on its own.

We tested three hypotheses. Hypothesis 1 was that, compared to those who use formal help, exclusive use of informal help would be associated with less severe mental disorder. This was supported. Those with a less severe score on the CIS-R, not having depression, and not having suicidal ideation were more likely to exclusively seek informal help. Informal help-seekers were also more likely to rate themselves as more healthy, and less likely to report longstanding illnesses or functional limitations due to emotional health.

Contrary to our second hypothesis, we found no evidence that higher social support was associated with exclusive use of informal help seeking. We did however find that those with social support seemed less likely to seek formal help on its own. This supports Woodward et al. [13] who found that those with larger social networks were more likely to use both formal and informal help amongst their African American and black Caribbean participants.

When we tested Hypothesis 3 and compared those who had exclusively used informal help and those who had sought formal help, we found no differences in socio-demographic factors (age, gender or ethnic group), when unadjusted and adjusted for clinical factors. With Hypothesis 4, in the unadjusted model, those in lower SES groups were found to be less likely to use informal help but this association was rendered non-significant when models were adjusted for clinical severity. These results suggest that it is not socio-demographic or socio-economic factors that drive informal or formal help-seeking, but clinical factors such as severity and complexity that do.

This study has some limitations. The survey took place in an urban area in London so that findings from this study may not be generalisable to all communities. This is an area with greater access to more services, because of the presence of a very large mental health trust. However, there is also likely to be a higher level of need and mental health difficulties because of the higher deprivation level of the area. A national survey showed that in London, there are slightly higher rates of stigma, as indicated by intended contact with a person with a mental illness [33] but that a greater reduction in stigma also occurred over time [34]. Given this, it is likely that these results would be generalizable to other urban areas but less so to rural areas. The ethnic population is also different from that in other areas, comprising more black Caribbeans and black Africans and fewer Asians. And while attempts were made to create adequate groupings, some cells (e.g. ethnic minority groups) were small. In terms of service use assessment, self-report may be open to recall bias; however, other studies have largely shown that self-reported service use shows reasonable agreement with other sources, including administrative records among those with measured mental health need [35]. The cross-sectional nature of the survey also limits our ability to understand the causal relationship between different types of help-seeking and the longitudinal patterns of access to help. The wording of the question may have affected responses as formal help related to ‘anxious or depressed or a mental, nervous or emotional problem’ whereas informal help related to ‘an emotional problem’.

The 4 help-seeking group categorization used in this study is similar to that previously used by Woodward and colleagues [13] in their study of African Americans and Black Caribbeans with lifetime mood, anxiety or substance misuse problems. They found 23% had used informal help only, 41% had used both informal and formal help, 14% used formal help only and 22% had sought no help. These figures are slightly different from our study in which fewer ‘cases’ sought either informal help only or no help at all. It is likely that the differences can be explained by the greater chronicity of problems and ethnic differences of their participants. In the UK, Rudell and colleagues [14] also found a pluralistic pattern of help-seeking with GP consultation occurring alongside informal help-seeking.

Work with adolescents [12] and ethnic minorities [13], indicates seeking informal help is often preferred when seeking help for mental disorders. Our study supported those findings; the more frequent users of informal help in this study were younger people and those from ethnic minority groups. In addition, we found employed people frequently used informal help.

We also found that different help-seeking patterns varied by ethnic group. Black Caribbeans used their friends more than family members and used formal help quite frequently. Asians were more likely to use their family, but not use formal help. Black Africans were more likely to seek help from a religious leader and be less likely to seek formal help. Rudell and colleagues [14] also found ethnic differences amongst Bangladeshi and black Caribbean and white British in the choice of help-seeking strategies. They found that both the Bangladeshi and Caribbean groups used spiritual forms of help more frequently. However, Bangladeshi participants used medical help more than the other 2 groups, but informal help less frequently.

In terms of future research, there is a potentially a large research agenda. A key question is from whom people seek informal help. From this study and that by Rudell [14] there are ethnic differences which merit further research. Following on from this, barriers and facilitators to seeking informal help need investigation. Possible factors may be gender, ethnic background, characteristics of social networks and emotional competence. In our study, men, older adults, those with low educational qualifications and low income were less likely to use informal help. Closer examination of the attitudes of these groups to informal help would be useful.

Pathways from informal help to formal help are extremely important particularly for severe problems. Where formal help is indicated, it has been suggested that informal help could act as a bridge to access help for mental disorders [36] or as an early intervention because formal resources are not always available [37]. A key question is what determines the decision to seek formal help. Possible triggers may be family support [13] and severity of problems [13]. However, there is also evidence that transferring from informal to formal help may not always occur. Lamb [17] found that barriers for ‘hard to reach’ groups, included withdrawal from wider social networks in order to protect their core identities in these communities, fearing difficulties would be labeled and stigmatized. These groups experienced the interface with primary care being difficult because of differences in values. There is also some anecdotal evidence that religious leaders offering informal support to people attending their church, may not always assist in the transition to formal help, often continuing to provide support themselves (Codjoe, personal communication).

The effectiveness of informal help is a crucial question. What constitutes informal help might be difficult to define, as by their nature, this help varies, and is not often sought regularly or consistently. It is by definition much more difficult to assess informal help because researchers are rarely present when the person with the mental health problem first approaches a friend or a member of the family. Less conventional designs may need to be used. Studies might need to be retrospective or if prospective, focus around a life problem (e.g. unemployment, traumatic incident) or with selected groups where longitudinal follow-ups are possible (e.g. college students). However, Pfeiffer et al. [38] conducted a meta-analysis of peer support interventions and found these were more effective than care as usual, and as effective as group CBT for depression. Additionally, individual characteristics of informal helpers and those helped merit investigation. One area may be the components of skilful informal help-giving. In a qualitative study, Griffiths et al. [39] found that the informal help can have many advantages such as social, emotional, informational and companionship support but there were also some disadvantages namely, stigma and inappropriate support. It would be also important to investigate characteristics of people who are easier and more difficult to help.

Finally, we found 26.3% of ‘cases’ did not seek any kind of help. This is consistent with findings by Oliver et al. who found that over 20% with more severe mental health problems were non-help-seekers. It is important to recognize that not all people with mental health problems will need formal help from services. Sareen et al. [40] found that 50% with diagnosable problems remitted without intervention at 3 year follow-up. Whiteford et al. [41] who examined untreated samples such as waiting list participants, estimated that over 53% with diagnosed depression would remit after 12 months and found that severity affected remission. Information about the use of informal help was not available in either study. It would be helpful to conduct a closer investigation of those who spontaneously remit to see if informal help has been relevant, and to whom.

### Service implications

Given how frequently informal help is used, much more attention should be paid to informal networks. Working with patients’ social networks could lead to more accessible and possibly better outcomes. Planning services around social networks of different ethnic and social groups could help better target services. Thus, offering help to families of Asian and older people with mental health problems could match the patterns for these groups whereas offering help to friends of black Caribbeans would be more natural for that group. It may be that once we better understand the role of informal and formal help for people in a population, then we would be in a better position to implement Kleinman’s [7] ideas about matching perceptions of problems, and then offering interventions that ‘fit’ better. Access for ‘hard to engage’ groups could include both raising awareness of services in primary care for these groups, as well as services bridging the ‘gap’ through understanding these people’s positions and perceptions better, resulting in a broader ‘treatment’ [17]. Involving primary care services in identifying these ‘hard to engage’ people has been attempted, although the numbers were small and the success limited [42]. A key issue about the use of informal help is how professionals feel about it as they do not always endorse this [4,5]. Nevertheless, informal help relates to the optimal mix of services proposed by the World Health Organisation (WHO) with self-care and informal community care being the bottom 2 layers prior to primary care services [6]. They propose that these informal services can be both helpful in preventing demands being made in the formal services, as well as be helpful when people are discharged from the formal services. While proposed with less developed countries in mind, this could be a useful model globally.

## Conclusions

This study has shown that informal help-seeking is an extremely important and commonly used process. Among the ‘cases’, 62.6% had sought informal help, whether on its own (33.6%) or together with formal help (29%) with 26.3% not having sought any help at all. Only 11.1% had sought formal help on its own. Many papers focus on the statistic that only a third of people with mental health problems seek formal help. To this statistic needs to be added another - that a third seek informal help only, which leaves only a third not seeking any help at all. If we are to harness informal help, then we urgently need to research its use, as well as its effectiveness.
